# A MCP1 fusokine with CCR2-specific tumoricidal activity

**DOI:** 10.1186/1476-4598-10-121

**Published:** 2011-09-24

**Authors:** Moutih Rafei, Jiusheng Deng, Marie-Noëlle Boivin, Patrick Williams, Shannon M Matulis, Shala Yuan, Elena Birman, Kathy Forner, Liangping Yuan, Craig Castellino, Lawrence H Boise, Tobey J MacDonald, Jacques Galipeau

**Affiliations:** 1 The Montreal Center for Experimental Therapeutics in Cancer, McGill University, Montreal, Canada; 2 The Institute for Research in Immunology and Cancer, Montreal University, Montreal, Canada; 3 Department of Hematology and Oncology, Emory University, 1365B Clifton Road, Clinic B, Atlanta, GA 30322, USA; 4 Department of Pediatrics, Winship Cancer Institute, Emory University, 1365B Clifton Road, Atlanta, GA, 30322, USA

## Abstract

**Background:**

The CCL2 chemokine is involved in promoting cancer angiogenesis, proliferation and metastasis by malignancies that express CCR2 receptor. Thus the CCL2/CCR2 axis is an attractive molecular target for anticancer drug development.

**Methods:**

We have generated a novel fusion protein using GMCSF and an N-terminal truncated version of MCP1/CCL2 (6-76) [hereafter GMME1] and investigated its utility as a CCR2-specific tumoricidal agent.

**Results:**

We found that distinct to full length CCL2 or its N-truncated derivative (CCL2 5-76), GMME1 bound to CCR2 on mouse lymphoma EG7, human multiple myeloma cell line U266, or murine and human medulloblastoma cell lines, and led to their death by apoptosis. We demonstrated that GMME1 specifically blocked CCR2-associated STAT3 phosphorylation and up-regulated pro-apoptotic BAX. Furthermore, GMME1 significantly inhibited EG7 tumor growth in C57BL/6 mice, and induced apoptosis of primary myeloma cells from patients.

**Conclusion:**

Our data demonstrate that GMME1 is a fusokine with a potent, CCR2 receptor-mediated pro-apoptotic effect on tumor cells and could be exploited as a novel biological therapy for CCR2^+ ^malignancies including lymphoid and central nervous system malignancies.

## Background

CC Chemokines and their cognate receptors are involved in the proliferation and metastasis of several tumors [1]. The CCL2/CCR2 axis is a direct example as highlighted by CCL2-driven proliferation and survival of hematological [2,3] and solid tumors [4,5]. Thus, inhibiting CCL2 or its receptor may allow a direct interference with tumor biology.

As an alternative to the development of neutralizing or antagonizing antibodies, our group has focussed on the engineering of bifunctional proteins borne from the fusion of two biologically distinct cytokines [6-12]. These fusokines have been shown to lead to novel unheralded pharmacological effects including potent, receptor-specific antitumor effects [6,11]. Interestingly, granulocyte macrophage-colony stimulating factor (GMCSF)-based fusokines may either lead to pro-inflammatory synergy or profoundly antagonistic properties depending on the influence played by the GMCSF moiety of the fusokine on the C-terminal partner signalling pathway.

**Figure 1:**
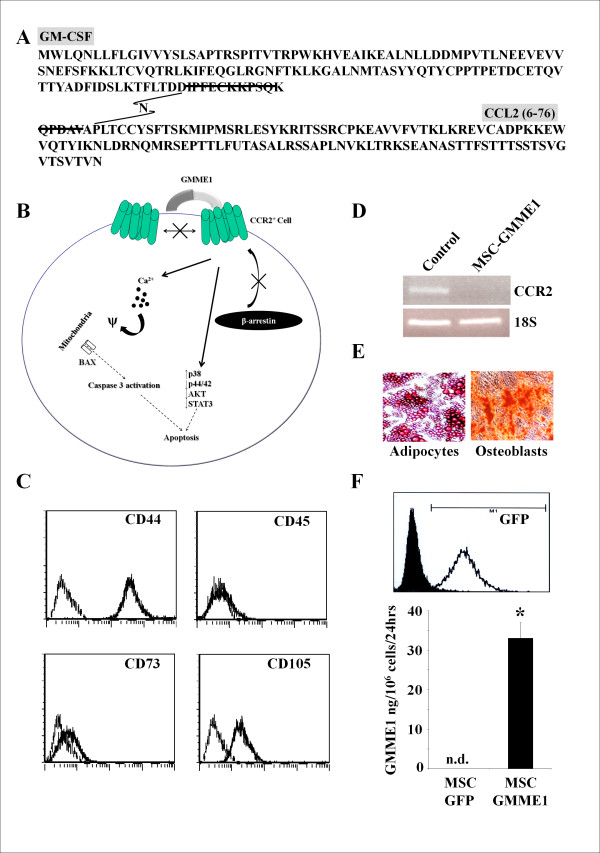
**Phenotype analysis**. **A**. The amino acid sequence of GMME1. **B**. GMME1 Mechanism of Action. GMME1 is capable of blocking CCR2 homodimerization and recruitment of β-arrestin. As a result, various biochemical responses take place such as an increase in p38 phosphorylation while p44/42, AKT, STAT3 are inhibited. In addition, a strong Ca^2+ ^influx s triggered leading to the activation of caspase 3 and apoptosis. **C**. The phenotype of expanded C57BL/6 MSCs was analyzed by flow cytometry for various cell surface markers. **D**. An RT-PCR analysis demonstrates that MSC do not express CCR2. **E**. MSC culture under adipogenic or osteogenic conditions leads to their differentiation. Photographs were taken under light microscopy using a Contax167MT camera (Kyocera) with a 400 ISO film attached to an Axiovert25 Zeiss microscope (Carl Zeiss) **F**. Following the retroviral transduction of MSC, the GFP expression levels was monitored by flow cytometry with GMME1 secretion level at 33 ng/10^6 ^cell/24 hrs as detected by ELISA (P < 0.05; n = 3/group).

The previously described GMME1 fusion protein, composed of mouse GM-CSF and truncated CCL2 (6-76) missing the first 5 N-terminal amino-acids, binds to CCR2 and initiates an aberrant signalling cascade which activates a pro-apoptotic response associated with calcium flux, dephosphorylation of STAT3 and decreased pAKT (Figure 1A) [10,11].

We here report its use as a cancer therapeutic agent targeting tumors expressing CCR2 and provide pre-clinical evidence that this pro-apoptotic fusokine could be of great interest as a lead compound of a new class of biological agents targeting CCR2-expressing malignancies.

## Methods

### Mice, cell lines and reagents

All mice used were 6-8 week old C57Bl/6 females (Jackson Laboratory, Bar Harbor, ME). All experimental protocols were approved by the animal ethic committee of the Lady Davis Institute of McGill University. The mouse T-cell lymphoma cell line EG.7 (EL4 cells transfected to express chicken ovalbumin), human medulloblastoma cell line Daoy, and the human myeloma cell line U266 were purchased from ATCC (VA, USA) and propagated according to manufacturer's instructions. The murine medulloblastoma cell line PS125 was established from medulloblastomas derived from the Smo/Smo transgenic mouse [13] and propagated in DMEM/F-12 supplemented with 10% FBS, 1% L-glutamine, 1% MEM non-essential amino acids, and 1% N-2 at 37°C in 5% CO_2_. Antibodies for CD19, CD44, CD45, CD73, CD105 and CD138 were purchased from BD Biosciences (San Diego, CA). Mouse recombinant CCL2 protein (CCL2 1-76), ELISA kits for mouse CCL2 and human IL6, anti-human CCR2 antibody, CCR2 primers, and Annexin-V/PI detection kits were purchased from R&D systems (Minneapolis, USA). Antibody for α-tubulin was purchased from Santa Cruz Biotechnology (Santa Cruz, CA). Antibodies for BAX, pSTAT3 and total STAT3 were purchased from Cell Signalling Technology (Danvers, MA). RNA extraction kit was purchased from Qiagen (Mississauga, ON, CANADA). Contigen was purchased from Bard Urological Division (Covington, GA, USA). The 5-76 variant (CCL2 5-76) of murine CCL2 was synthesized from Genecust (Dudelange, Luxembourg). The monocyte enrichment kit was purchased from StemCell Technologies (Vancouver, Canada).

### Isolation and characterization of mouse mesenchymal stromal cells

Whole bone marrow was harvested by flushing femurs and tibias bones of female CCL2^-/- ^C57BL/6 mice with DMEM. Collected cells were plated and cultured until the appearance of a homogeneous polyclonal population of mesenchymal stromal cells (MSC). The plasticity of isolated MSCs was tested as previously reported [14]. For CCR2 expression on MSC, RT-PCR was performed on extracted RNA using purchased primers.

### Engineering CCL2^-/-^MSC to express GMME1

We have previously demonstrated that wild-type MSCs could generate in a paracrine fashion truncated CCL2 (5-76) capable of antagonizing CCR2-expressing cells [15]. Therefore, CCL2^-/-^MSCs were used in this study to avoid any confounding effects arising from endogenous MSC production of CCL2 and derivatives. The generation and concentration of green fluorescent protein (GFP) or GMME1 retroparticles using the bicistronic AP2 vector were generated as previously reported [6]. The level of GMME1 expression was analyzed through the assessment of GFP by flow cytometry, while GMME1 secretion level in the harvested DMEM conditioned medium was quantified using a CCL2 ELISA kit. Alternatively, GMME1 protein was purified from the conditioned medium with an affinity column (Pierce Biotechnology, Rockford, USA, Kit # 26148) loaded with anti-mouse GM-CSF antibodies (R&D, Minneapolis, USA) by following the instruction described in the kit. The purified GMME1 protein was dialyzed with fresh DMEM medium, and concentrated for use.

### Biochemical analyses

To test the proliferative property of GMME1, the mouse lymphoma EG7 or human multiple myeloma U266 cell lines were plated at a density of 10^5 ^cells/well in a 96-well plate and treated with increasing concentrations of cytokines for 48 hours. The reaction was read at 570 nm after adding 20 μL of 3-(4,5-dimethylhiazol-2-yl)-2,5-diphenyltetrazolium bromide (MTT). For Apoptosis analysis, the mouse EG7 or human U266 cell lines were cultured for 48 hrs with equimolar concentrations (1.5 pmol) of CCL2 (1-76), CCL2 (5-76), or GMME1 then analyzed by PI/Annexin-V. WT and CCR2^-/- ^monocytes were enriched to 90% purity using negative selection following the bone marrow flush of femurs and tibias. Purified cells were then cultured for 48 hrs in control or GMME1 supernatant. A cell-killing assay was also performed on two medulloblastoma cell lines PS125 (mouse-derived) and Daoy (human-derived), treated with or without GMME1 for 48 hrs and cell death measured by flow-cytometry using PI and Annexin-V. Alternatively, Daoy cells were also treated with GMME1 in conditioned medium or affinity-purified GMME1 protein, and the cell growth was assessed by MTT assay. Western blot was performed on the lysate derived from treated cell lines probed with anti-BAX antibodies, or anti-pSTAT3 or anti-STAT3 antibodies. IL-6 secretion by U266 was quantified with ELISA, following the different cytokine treatments. For signalling analysis, a sandwich ELISA for mouse/human STAT3 was performed.

### Cancer induction and treatments

To study the locoregional effect of GMME1 on tumor development, 2 × 10^6 ^MSC-GFP were co-implanted with 10^6 ^EG7 cells subcutaneously (sc) in immunocompetent C57Bl/6. For systemic efficacy of the fusokine, 10^6 ^EG7 cells were injected sc in immunocompetent C57Bl/6 mice on one side, and an sc implant of contigen-embedded gene-engineered MSCs (2 × 10^6 ^cells per implant) was injected on the opposite flank as previously described [14]. Tumor appearance and volume were assessed every 48 hrs. To investigate the levels of circulating GMME1 in treated mice, the sera were collected at week 3 post-implantation of the neo-organoid and screened by CCL2 ELISA to detect the CCL2 moiety of the fusokine according to manufacturer's instructions.

### GMME1-induced apoptosis of primary myeloma cells from patients

Bone marrow aspirates from consenting myeloma patients (n = 5) were processed as previously described [16]. The IRB protocol was approved by Emory University. White blood cells in the marrow were isolated with lymphocyte separation medium (Mediatech Inc., Manassas, VA), and stained with anti-human CD38 (PE), CD45 (APC-Cy7), CD138 (FITC) (BD Bioscience, San Jose, CA), or CCR2 (PerCP) (Biolegend, San Diego, CA) antibodies or isotype controls (1:100 dilution). CD38^+^CD45^-^CD138^+ ^cells were considered as myeloma cells [16]. Alternatively, white blood cells (10^6 ^cells/ml) were cultured in RPMI 1640 medium with 10% FBS in presence of GMME1 (20 ng/ml). Condition medium without GMME1 served as control. After 48-hour culture, the cells were harvested and stained with anti-human CD38, CD45 antibodies, and Annexin. FACS analysis was performed on a BD FACSCanto II flow cytometer. Data were analyzed with FlowJo 9.1 software.

### Statistical analyses

*P *values were calculated using the ANOVA test, or paired t-test in Figure Six F.

## Results

### Gene engineering of MSC as GMME1 biofactories

GMME1 is a fusion cytokine consisting of granulocyte macrophage colony stimulating factor (GMCSF) in tandem with N-terminal truncated monocyte chemotactic protein-1 (MCP1 6-76) whose design and biochemical analysis were previously reported and summarized in Figure 1A-B. We have also previously demonstrated that gene- modified MSC can serve as robust cytokine biofactories *in vitro *and *in vivo *[10,11,14,17-21]. We here sought to exploit this platform to produce recombinant GMME1 *in vivo*. To obviate any confounding effect arising from naturally produced CCL2 from MSC, MSC isolated from CCL2^-/-^C57Bl/6 mice were engineered a polyclonal population to produce secreted GMME1 as we previously published [10,11]. MSC expressing GMME1 are CD44, CD73 and CD105 positive with no detectable CD45 (Figure 1C) or CCR2 (Figure 1D) while retaining their capacity to differentiate into adipocytes or osteoblasts (Figure 1E). Supernatant analysis of these cells revealed a GMME1 secretion level at 30 ng per 1 million cells every 24 hrs (Figure 1F). The conditioned media (CM) from these cells was used as a source of GMME1 for *in vitro *experiments and the engineered MSC were lately used as *in vivo *cell factories for drug delivery.

### GMME1 is tumoricidal to CCR2^+ ^murine EG7 lymphoma

**Figure 2:**
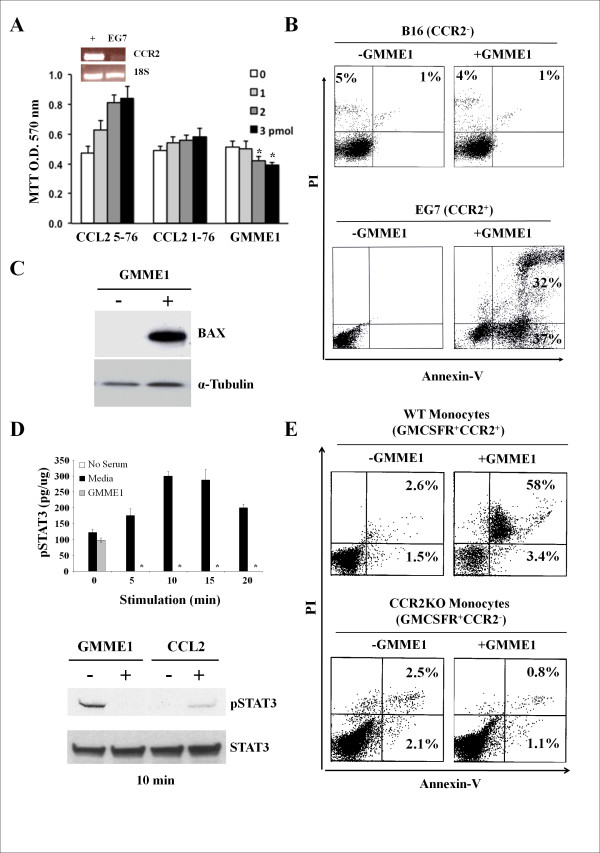
**Pharmacological properties of GMME1 on EG7 tumor cells**. **A**. Following the confirmation that EG7 cells express CCR2 by RT-PCR, 10^5 ^EG7 cells/well were cultured for 48 hrs in presence of increasing amounts of CCL2 5-76, CCL2 1-76 or GMME1 and the proliferative response measured by MTT. CCL2 5-76, and to a lesser extent, CCL2 1-76 were capable of inducing the proliferation of EG7 cells as opposed to GMME1 (P < 0.05; n = 6/group). **B**. Following the addition of 1.5 pmol of GMME1 on EG7 cells for 48 hrs (lower panel), a PI/Annexin-V co-staining demonstrates that GMME1 leads to apoptosis induction (32% dead cells). None of the B16 cells, which are CCR2 null, were affected by the addition of GMME1. **C**. EG7 cells cultured with GMME1 for 48 hrs induce *de novo *expression of the pro-apoptotic BAX protein. **D**. Following the stimulation of EG7 cells for different time points, cell lysate was analysis by a pSTAT3 ELISA (P < 0.05; n = 6/group). The experiments were repeated using the 5 min time point, then lysate was probed by WB. Total STAT3 was used as loading control. **E**. WT or CCR2^-/- ^monocytes were purified and cultured with 1.5 pmol of GMME1 for 48 hrs before a PI/Annexin-V co-staining. Even though 58% of WT monocytes died, no major cell death was detected with CCR2^-/- ^monocytes.

Since the truncated CCL2 domain of GMME1 is only active on CCR2 [10,11], we determined the pharmacological effect of the fusokine on the EG7 mouse lymphoma cell line known to express CCR2 (Figure 2A). No proliferative response was observed upon GMME1 treatment in contrast to that seen with the use of CCL2 5-76 (Figure 2A). Since we have previously shown that only GMME1 induces apoptosis on CCR2^+ ^cells, PI/Annexin-V co-staining was performed on GMME1-treated EG7 and revealed 30% cell death following 48 hr treatment (Figure 2B) associated with *de novo *expression of the pro-apoptotic BAX protein (Figure 2C). CCR2 is known to induce STAT3 phosphorylation; a signalling molecule involved in survival, proliferation, angiogenesis as well as immunosuppression [22]. Thus, GMME1 was tested for its effect on STAT3 phosphorylation. A complete blockade was obtained within 5 min following treatment, an observation that was confirmed by Western Blot (Figure 2D). To exclude any possible involvement of the GMCSF moiety of the fusokine in inducing cell death, monocytes were purified from CCR2^-/- ^mice bone marrow and treated with GMME1 versus control. No cell death was detected on CCR2^-/- ^monocytes as opposed to WT control cells (Figure 2E).

### GMME1 leads to EG7 growth suppression *in vivo*

**Figure 3:**
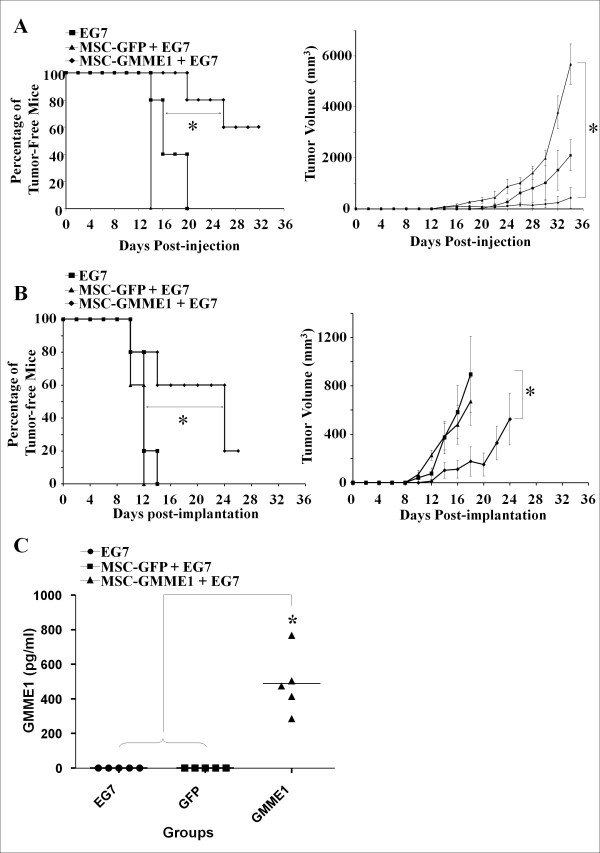
**Anti-tumor effect of GMME1-expressing CCL2^-/-^MSC**. **A**. Subcutaneous injection of immuncompetent C57Bl/6 mice (n = 5/group) with 10^6 ^EG7 cells alone or in combination with 2 × 10^6 ^MSC-GFP leads to the development of large tumors in all mice. The admixture of GMME1-expressing MSC with EG7 tumor cells leads to a significant delay in EG7 growth with 60% of mice that remained tumor-free for over a month (*P < 0.05). **B**. To assess the growth of EG7 cells in mice implanted with a neo-organoid expressing GFP vs the GMME1 fusokine, immunocompetent C57Bl/6 mice (n = 5/group) were injected on one flank with contigen containing 2 × 10^6 ^MSC engineered to express GFP or GMME1 followed by 10^6 ^EG7 cells on the opposite flank. Tumor volume and appearance was assessed every 48 hrs. A significant delay in tumor growth was noticed (*P < 0.05). **C**. Three weeks post-contigen implantation, mice were bled and the sera tested using the CCL2 ELISA kit for GMME1. All mice implanted with MSC expressing the fusokine GMME1 showed detectable levels as opposed to control MSC-GFP or mice implanted with EG7 cells only (*P < 0.05).

To assess the anti-tumor properties of GMME1 *in vivo*, we proceeded with the subcutaneous co-implantation of 2 × 10^6 ^MSC-GMME1 admixed with 10^6 ^EG7 lymphoma cells in immunocompetent C57Bl/6 mice and monitored tumor growth relative to controls over time. All mice implanted with MSC-GFP and EG7 developed tumors by day 14 (Figure 3A upper left panel) with significantly larger volumes when compared to EG7 tumors cells alone (Figure 3A upper right panel). In contrast, when GMME1-expressing MSC were coimplanted with EG7 cells, a significant delay in tumor growth was observed with 60% tumor-free mice (Figure 3A). A more clinically relevant approach however, consists of delivering GMME1 systemically rather than peritumorally. Therefore, immunocompetent C57Bl/6 mice were implanted subcutaneously with GMME1-secreting MSC on one flank of the animal and the tumor cells on the opposite flank. A substantial antitumor effect was obtained with GMME1 since 20% of mice were tumor-free with a significant tumor growth delay up to 3 weeks post-implantation of the GMME1-producing MSC (Figure 3B). This therapeutic effect correlates with the plasma levels of GMME1 at this time point (Figure 3C). Mice treated with GMME1 did not display evident off target toxicity as ascertained by normal weights and behaviour (data not shown).

### GMME1 is tumoricidal to human CCR2^+ ^U266 myeloma cells

**Figure 4:**
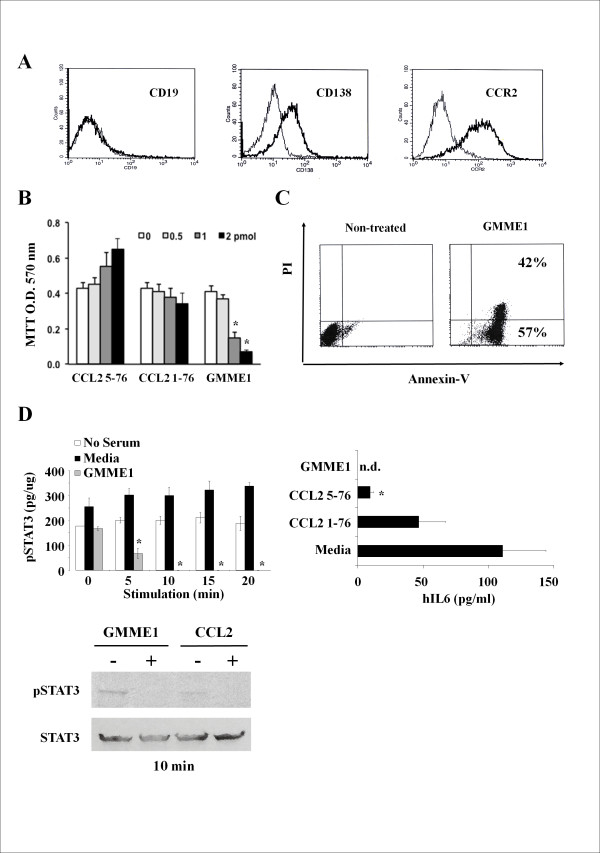
**Pharmacological properties of GMME1 on human U266 tumor cells**. **A**. U266 cells were analyzed by flow cytometry and were negative for the expression of CD19 while CD138 and CCR2 were detected. **B**. 10^5 ^U266 cells were cultured with increasing amounts of CCL2 5-76, CCL2 1-76 or GMME1 and the proliferative response measured by MTT. CCL2 5-76 was capable of inducing U266 proliferation whereas GMME1 completely suppressed the proliferative response (P < 0.05; n = 6/group). **C**. Using 1.5 pmol of GMME1 on U266 cells for 48 hrs, a PI/Annexin-V co-staining demonstrates that GMME1 leads to apoptosis (42% cell death). **D**. A similar set-up was used for the assessment of STAT3 activation on U266 cells. Following the stimulation of U266 cells using different time points, cell lysate was analysis by a pSTAT3 ELISA. Since STAT3 is inhibited as of 10 min following GMME1 addition on U266 cells, the experiment was repeated at this time point then the lysate was probed by WB. Total STAT3 was used as loading control. To further confirm the inhibitory effect of GMME1 on these cells, the U266 conditioned-media was collected following 48 hrs post-treatment with the different test conditions and analyzed using a human IL-6 ELISA kit. No detectable levels of human IL6 could be observed in the GMME1 group as opposed to the remaining test conditions (*P < 0.05; n = 6/group).

Mouse CCL2 is biologically active on human CCR2-expressing cells [23]. In light of this inter-species permissiveness, we assessed the pharmacological properties of mouse GMME1 on the human multiple myeloma cell line U266, a CD19^-/- ^human myeloma cell line shown to express the plasma cell marker CD138 and CCR2 (Figure 4A). U266 cells proliferated in a dose-dependent manner using control N-terminus truncated CCL2 5-76 whereas GMME1 led to a substantial proliferation blockade (Figure 4B). To further confirm this observation, PI/Annexin-V analysis following 48 hrs GMME1 treatment led to 40% cell death by apoptosis (Figure 4C). U266 growth and proliferation depends on the autocrine secretion of human IL6, which leads to pSTAT3 [22]. Since we have previously shown that GMME1 inhibits STAT3 phosphorylation in EG7 lymphoma cells, we assessed the level of STAT3 activation first by ELISA at different time points and documented a complete loss of activation following 10 min of GMME1 treatment, an observation that was confirmed by immunoblot (Figure 4D left panels). These data correlate with the loss of human IL6 secretion by U266 (Figure 4D right panel) due to cell death induced by GMME1.

### GMME1 is tumoricidal to mouse and human CCR2^+ ^medulloblastoma cells

**Figure 5:**
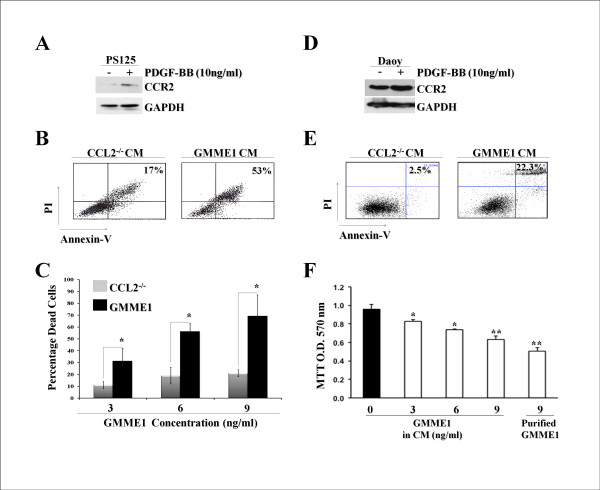
**Pharmacological properties of GMME1 on CCR2^+ ^medulloblastoma cells**. **A**. Confirmation of CCR2 expression on mouse medulloblastoma cell line PS125. **B**. PS125 cells were seeded in 6-well plates (10^4 ^cells/well) and cultured with CCL2^-/- ^or GMME1 CM. After 48 hrs, apoptosis was measured by Annexin-V/PI staining. **C**. To further see a dose-response effect, the same experiment was repeated using 3 different concentrations and cell death was analyzed (P < 0.05; n = 3/group). **D**. Human medulloblastoma cells (Daoy) were analyzed by WB to confirm the presence of CCR2 on cell surface. **E**. Human medulloblastoma cells were treated with GMME1 as described in **B**. Cell apoptosis was measured by Annexin-V/PI staining after 48 hrs culture. **F**. Human medulloblastoma cells were cultured in presence of GMME1 in condition medium or affinity-purified GMME1 protein. Cell growth was measured by MTT assay. (*P < 0.05; **P < 0.01; n = 3/group).

Human glial tumors are known to express CCR2, though the biological significance of this observation is unknown [24]. We tested whether medullobalstoma cell lines also possess this feature. We found that both mouse (PS125) and human (Daoy) medulloblastoma cell lines expressed CCR2, and this expression was enhanced by PDGF, a known pro-oncogenic stimulus for this malignancy as shown in Figure 5A and 5D, respectively [13]. We tested whether GMME1 was tumoricidal to these medulloblastoma cell lines and observed that a substantial fraction of tumors treated with GMME1 died by apoptosis as opposed to control groups (53% vs 17% for the mouse cell line in Figure 5B, and 22.3% vs 2.5% for the human cell line in Figure 5E). This pro-apoptotic effect was also shown to be GMME1 dose-dependent (Figure 5C and 5F respectively). Moreover, we confirmed that affinity-purified GMME1 protein possesses strong tumoricidal activity on human medulloblastoma cells (Figure 5F).

### GMME1 induced apoptosis of primary multiple myeloma cells

**Figure 6:**
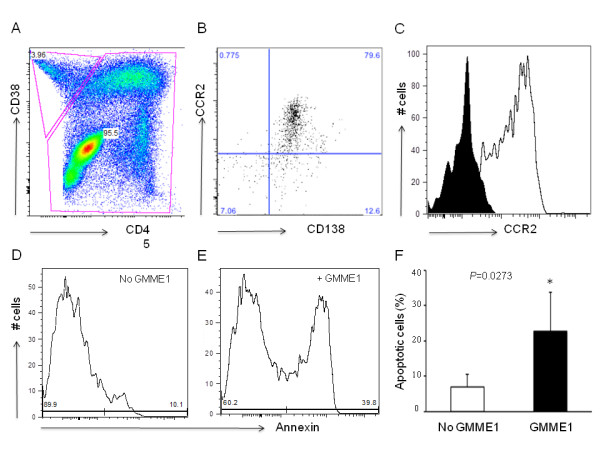
**GMME1 induced apoptosis of primary myeloma cells from patients**. **A**. White blood cells were isolated from bone marrow aspirates from patients with myeloma, and stained with anti-human CD38, CD45 and CD138 antibodies. CD38^+^CD45^-^CD138^+ ^cells were considered as myeloma cells. **B-C**. CCR2 highly expressed on myeloma cells (black field is antibody isotype control). **D-E**. Isolated lymphocytes were cultured in absence (**D**) or presence (**E**) of GMME1, and the apoptotic myeloma cells were determined by annexin staining (**D **and **E**). **F**. Percentage of apoptotic myeloma cells from patients was presented as the mean+/-SD (*P < 0.05; n = 5).

Human multiple myeloma is a clonal plasma cell characterized by resistance of apoptosis by expression of a panel of anti-apoptotic molecules [16]. We have previously showed that the multiple myeloma cell line U266 expressed CCR2 and is susceptible to GMME1-induced apoptosis (Figure 4A-C). Thus, we predict that GMME1 protein would trigger apoptosis in primary myeloma cells collected from consenting patients. Profiling of CD38^+^CD138^+^CD45^- ^myeloma cells (Figure 6A-B) isolated from patients by FACS confirmed the expression of the chemokine receptor CCR2 (Figure 6B-C). Subsequent treatment of primary myeloma cells with GMME1 (Figure 6E-F) for 48 hours *in vitro *led to substantial and significant apoptosis in comparison with the condition medium control (Figure 6D, F).

## Discussion

Interfering with the CCL2/CCR2 ligand/receptor pathway may be of meaningful use in cancer therapy and we here tested whether the CCR2-selective, pro-apoptotic effects of GMME1 we observed previously [10,11] could be utilized in such a setting. We tested the effect of GMME1 on the CCR2-expressing murine EG7 lymphoid and the human U266 myeloma cell lines *in vitro*. We found that GMME1 induces their death as previously observed in autoreactive immune cells [10,11]. A common mechanistic denominator is suppression of phosphorylation of STAT3 and induction of pro-apoptotic effectors such as BAX (none of the remaining Bcl-2 pro-apoptotic molecules were induced). We may speculate that the GMME1/CCR2 interaction leads to recruitment of a GPCR-linked phosphatase or activates an alternate signalling pathway interfering or competing with STAT3 activation. We have previously shown that delivery of GMME1 via a gene-enhanced cellular platform to mice ill with EAE or arthritis, led to immune suppression and clinical remission and we here found that the same platform could serve to treat mice implanted with CCR2^+ ^EG7 lymphoma. These data demonstrate that GMME1's tumoricidal properties can be replicated *in vivo *as well, demonstrating that its pro-apoptotic effects are unaffected by tumor/host interactions.

Intriguingly, non-hematological epithelial malignancies, such as prostate cancer, have been found to be addicted to CCL2 for their survival and malignant behaviour [25,26]. Furthermore, an astonishingly high proportion of primary human glial tumors overexpress CCR2 (whilst normal brain structures do not), suggesting possibly a similar mechanistic bonding to CCL2 [24]. We here demonstrate that medulloblastoma cells of both murine and human origins express CCR2 and are susceptible to GMME1-mediated apoptosis *in vitro*. There have been meaningful attempts at targeting the CCL2/CCR2 pathway for cancer therapy. For example, it was believed that neutralizing CCL2 would be of beneficial potential for the control of CCR2-expressing tumor cells. Unexpectedly, the systemic administration of anti-CCL2 antibodies in prostate cancer mouse models only slightly attenuated the proliferation of tumor cells likely due to the "rescue" of the CCR2^+ ^tumor cells by alternate chemokine ligands [27]. As an alternative, antagonizing CCR2 was suggested as an approach with wider applicability for cancer therapy. A group has investigated the use of a dominant negative CCL2 construct lacking 2-8 amino acids at its N-terminus (7ND) targeting CCR2 in a melanoma mouse model [28]. This study demonstrated modest effects *in vivo *most likely because of low expression levels and non-efficient delivery method [28].

In contrast, GMME1 is radically distinct in its tumoricidal CCR2-targeted function since it is not simply a decoy or passive dominant negative, but rather is an active ligand leading to receptor-mediated activation of apoptosis. In essence, GMME1 behaves as an entirely novel chemokine, co-opting CCR2 signaling machinery to compel CCR2^+ ^malignant cells to enter apoptosis. This mechanism of action is attractive as a non-cross-resistant cancer-killing pathway that may complement current therapies for resistant or relapsing CCR2^+ ^hematological malignancies, pediatric medulloblastoma or human multiple myeloma, and other CCR2^+ ^tumors as well. The fact that GMME1 protein can significantly induce cell death of CCR2^+ ^primary myeloma from patients indicates its potential clinical utility.

It should be noted however that CCR2-mediated *in vitro *killing of tumor cells does not exclude a possibility that *in vivo *anti-tumor activity of GMME1 is mediated at least in part by killing of CCR2-positive cells within the tumor microenvironment such as macrophages, which are known to express CCR2 and to support tumor growth. We have previously demonstrated [10] that CCR2^+ ^macrophages harvested from C57BL/6 mice and exposed to GMME1 died by apoptosis 24 h later as shown by activation of caspase-3 as well as annexin-V/PI co-staining. Such macrophage-depleting capacity of the fusokine does not involve the GMCSF moiety since CCR2^-/- ^monocytes expressing the GMCSFR do not die following GMME1 treatment. This set of data suggests that GMME1 can theoretically deplete macrophages in tumour that can potentially play a role in angiogenesis and thus promoting tumor growth.

## Conclusions

In conclusion, we have previously demonstrated that the use of GMME1 fusokine could be of significant therapeutic value for depletion of CCR2 autoreactive lymphomyeloid cells. We here further demonstrate that GMME1 also represents a conceptually novel biological approach for eradication of CCR2-expressing malignant cells without noticeable off-target toxicity to the host. The use of innovative chimeric CC-ligand fusokines could serve as a prototype strategy seeking to selectively deplete cancers whose proliferation and survival depends upon CCR-driven signalling.

## List of abbreviations

BAX: Bcl-2-associated × protein; CCL2: C-C motif Ligand 2; CCR2: C-C motif Receptor 2; GFP: Green Fluorescent Protein; CM: conditioned-media; GMCSF: granulocyte-macrophage colony-stimulating factor; GMME1: GMCSF-MCP1 fusion protein; EAE: Experimental Autoimmune Encephalomyelitis; ELISA: Enzyme-linked immunosorbent assay; MCP1: monocyte chemotactic protein-1; MSC: Mesenchymal Stromal Cells; MTT: 3-(4,5-dimethylhiazol-2-yl)-2,5-diphenyltetrazolium bromide; PI: Propidium Iodine; STAT3: Signal transducer and activator of transcription 3; 7ND: Decoy chemokine

## Competing interests

The authors declare that they have no competing interests.

## Authors' contributions

MR conceived designed and coordinated the study, performed *in vitro *and all *in vivo *experiments. JD designed and carried out partial *in vitro *experiments. MB phenotyped MSC and performed RT-PCR assays. PW performed apoptosis analysis on CCR2KO macrophages. SMM performed myeloma patient sample processing, cell profiling and apoptosis assay. SY participated in preparing CCR2KO macrophages. EB participated in *in vivo *experiments. KF coordinated and participated in all *in vivo *experiments. LY performed western blots on CCR2^+ ^cell lines. CC conceived and participated in experiments involving human CCR2^+ ^cells. LHB participated in the design and coordination of human myeloma samples. TM participated in the design and coordination of human cell lines experiments. JG conceived of the study, and participated in its design and coordination and helped to draft the manuscript. All authors read and approved the final manuscript.
